# RETRACTION: lncRNA‐PDPK2P Promotes Hepatocellular Carcinoma Progression Through the PDK1/AKT/Caspase 3 Pathway

**DOI:** 10.1002/1878-0261.70044

**Published:** 2025-05-05

## Abstract

**RETRACTION**: W. Pan, W. Li, J. Zhao, Z. Huang, J. Zhao, S. Chen, C. Wang, Y. Xue, F. Huang, Q. Fang, J. Wang, D. Brand, and S. G. Zheng, “lncRNA‐PDPK2P Promotes Hepatocellular Carcinoma Progression Through the PDK1/AKT/Caspase 3 Pathway,” *Molecular Oncology* 13, no. 10 (2019): 2246–2258, https://doi.org/10.1002/1878-0261.12553.

The above article, published online on 01 August 2019 in Wiley Online Library (wileyonlinelibrary.com), has been retracted by agreement between the journal Editor‐in‐Chief, Kevin Ryan; FEBS Press; and John Wiley & Sons Ltd. A third party reported to the journal that the one of the P‐PDPK2P tumor images in Figure 2E appeared to exceed 2 cm in diameter, which exceeds the maximum size recommended by ARRIVE guidelines. Further investigation by the journal and the publisher also found evidence that the second and third P‐NC mice in Figures 2E and 2F appeared to be the same mouse, although both were reported as having received different treatments. The authors responded to an inquiry by the publisher and provided original data. An evaluation of these data by the publisher further determined that there were several discrepancies between what was shown in the original data and what was reported in the published article. The authors stated that no mice had been reused between the images in Figures 2E and 2F. The authors also confirmed that one tumor image in Figure 2E exceeded 2 cm, but that the research was performed at the Third Affiliated Hospital at the Sun Yat‐sen University and was in compliance with China's “Guideline for Welfare and Ethical Review of Laboratory Animals” (GB/T 35892‐2018), which sets no tumor diameter limits for nude mice.

The parties have determined that the authors violated ARRIVE guidelines governing the maximum size of tumor samples, which had been adopted by the journal at the time of submission. The parties have also determined that there is overwhelming evidence that two mouse subjects were reused in Figures 2E and 2F but were presented as having received different treatments. There were several other discrepancies between the original data provided by the authors and those presented in the published article. The retraction has been agreed to because the article did not conform to the ethical guidelines of the journal at the time of submission and because there is significant evidence that some data included in the article were inaccurate or misreported, which fundamentally compromises the editors' confidence in the conclusions presented. The authors did not indicate their agreement with the retraction.

**RETRACTION**: W. Pan, W. Li, J. Zhao, Z. Huang, J. Zhao, S. Chen, C. Wang, Y. Xue, F. Huang, Q. Fang, J. Wang, D. Brand, and S. G. Zheng, “lncRNA‐PDPK2P Promotes Hepatocellular Carcinoma Progression Through the PDK1/AKT/Caspase 3 Pathway,” *Molecular Oncology* 13, no. 10 (2019): 2246–2258, https://doi.org/10.1002/1878-0261.12553.

The above article, published online on 01 August 2019 in Wiley Online Library (wileyonlinelibrary.com), has been retracted by agreement between the journal Editor‐in‐Chief, Kevin Ryan; FEBS Press; and John Wiley & Sons Ltd. A third party reported to the journal that the one of the P‐PDPK2P tumor images in Figure 2E appeared to exceed 2 cm in diameter, which exceeds the maximum size recommended by ARRIVE guidelines. Further investigation by the journal and the publisher also found evidence that the second and third P‐NC mice in Figures 2E and 2F appeared to be the same mouse, although both were reported as having received different treatments. The authors responded to an inquiry by the publisher and provided original data. An evaluation of these data by the publisher further determined that there were several discrepancies between what was shown in the original data and what was reported in the published article. The authors stated that no mice had been reused between the images in Figures 2E and 2F. The authors also confirmed that one tumor image in Figure 2E exceeded 2 cm, but that the research was performed at the Third Affiliated Hospital at the Sun Yat‐sen University and was in compliance with China's “Guideline for Welfare and Ethical Review of Laboratory Animals” (GB/T 35892‐2018), which sets no tumor diameter limits for nude mice.

The parties have determined that the authors violated ARRIVE guidelines governing the maximum size of tumor samples, which had been adopted by the journal at the time of submission. The parties have also determined that there is overwhelming evidence that two mouse subjects were reused in Figures 2E and 2F but were presented as having received different treatments. There were several other discrepancies between the original data provided by the authors and those presented in the published article. The retraction has been agreed to because the article did not conform to the ethical guidelines of the journal at the time of submission and because there is significant evidence that some data included in the article were inaccurate or misreported, which fundamentally compromises the editors' confidence in the conclusions presented. The authors did not indicate their agreement with the retraction.

